# Supplementary material for: Global redox proteome and phosphoproteome analysis reveals redox switch in Akt
Source: Nat Commun. 2019 Dec 2;10:5486. doi: 10.1038/s41467-019-13114-4 (PMC6889415; doi:10.1038/s41467-019-13114-4)
Supplement: Supplementary file 1 — Supplementary Information [file 41467_2019_13114_MOESM1_ESM.pdf]

Global redox proteome and phosphoproteome analysis reveals redox switch in Akt

Su et al.

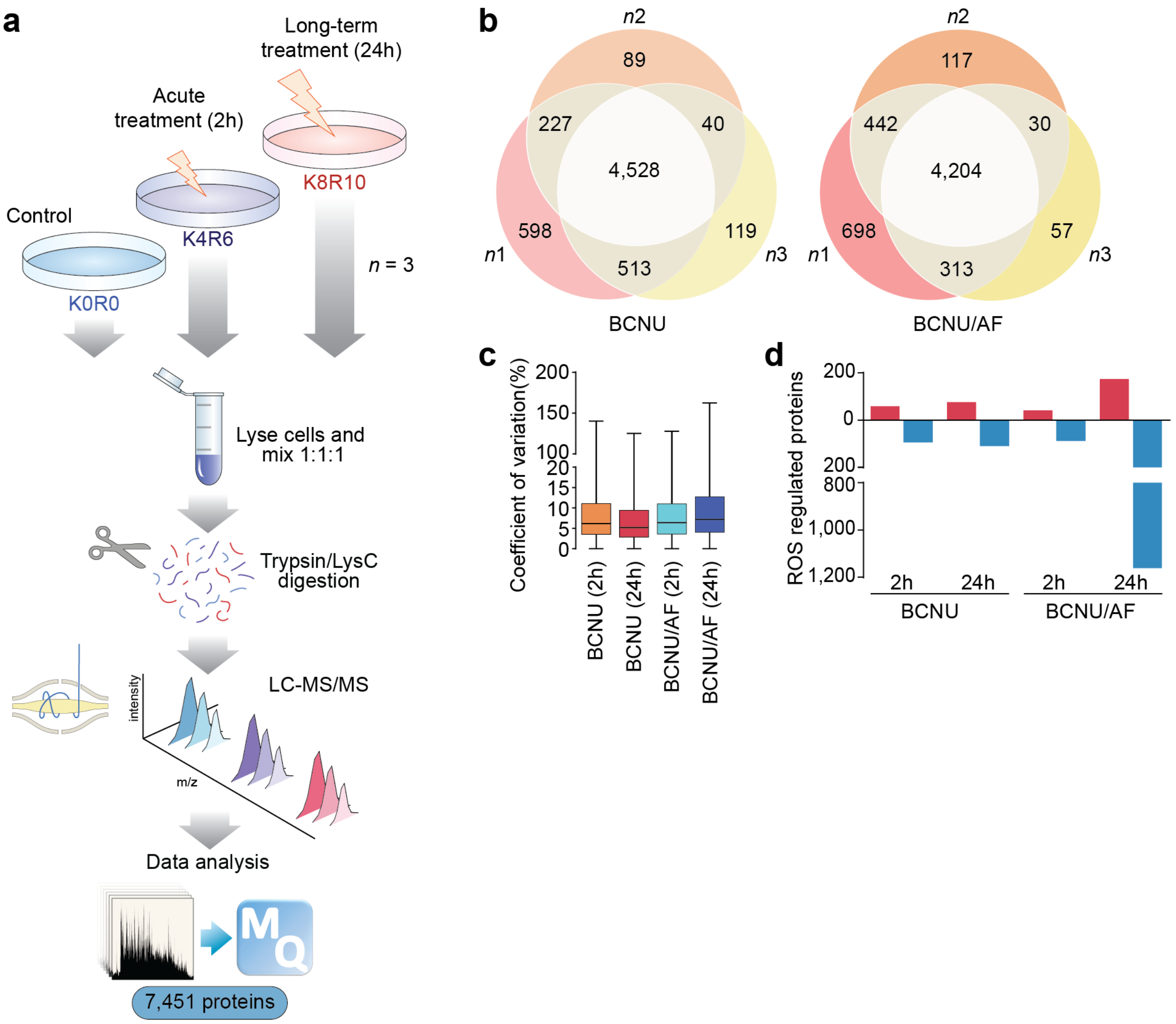

Supplementary Figure 1. Total proteomic analysis of adipocytes. (a) Experimental design of the total proteomic analysis of BCNU or BCNU/AF treated adipocytes. (b) Summary of the quantified total proteome. (c) Coefficient of variation of the quantified total proteome. Boxes capture lower quartile and upper quartile with median displayed as horizontal line in the middle. (d) Summary of oxidative stress-regulated total proteome in adipocytes with or without BCNU or BCNU/AF treatment.

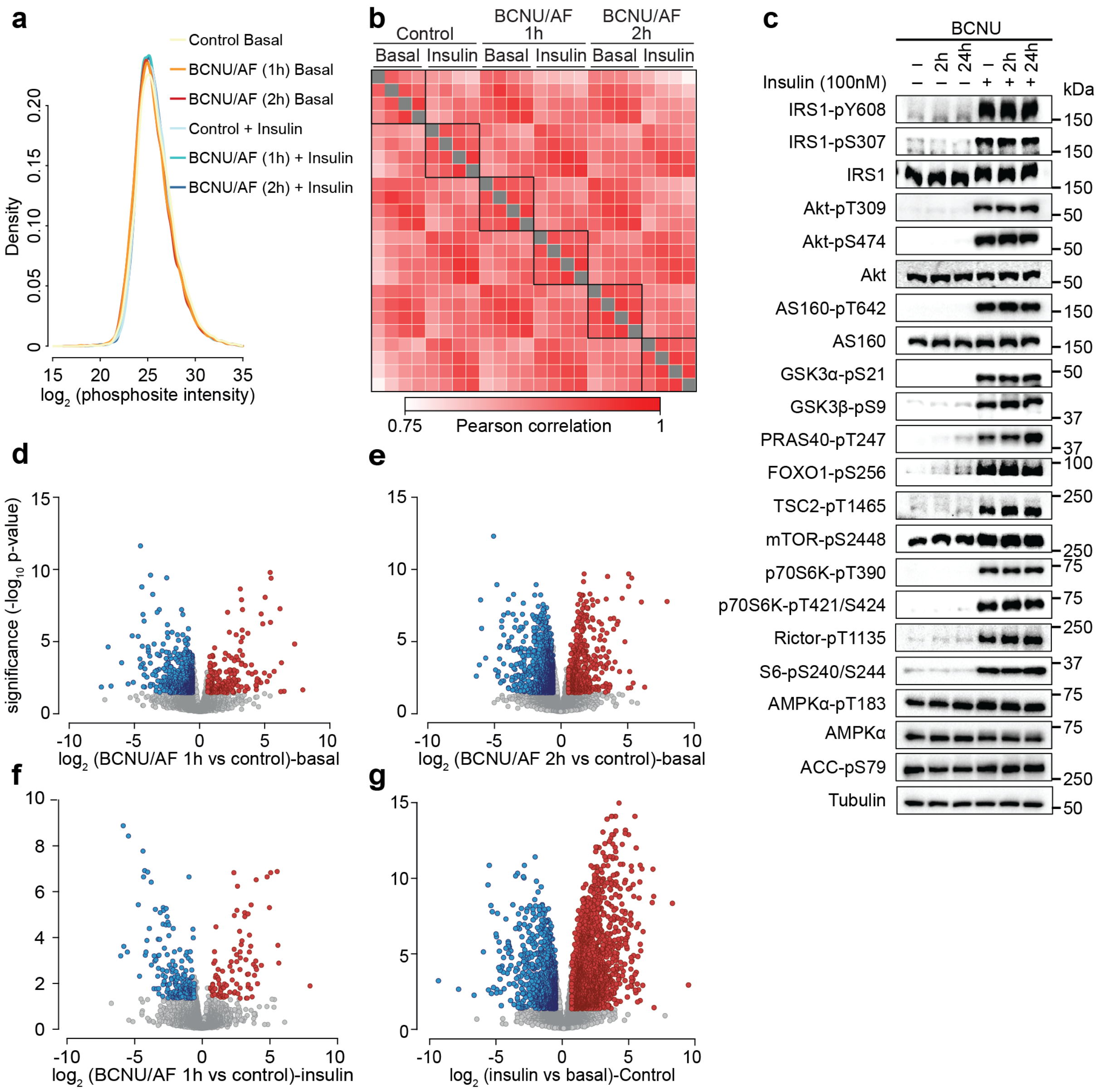

Supplementary Figure 2. Quality control analysis and differential expression analysis of the phosphoproteome. (a) Relative phosphosite intensities of the quantified phosphoproteome. (b) Pearson correlation coefficient of the quantified phosphoproteome. (c) Immunoblotting of key phosphosites in Akt, mTOR, and AMPK signalling pathways. Adipocytes were treated with BCNU for 2 or 24 h, serum starved and stimulated with insulin (100 nM, 20 min). The samples were assessed by immunoblotting with indicated antibodies. (d-g) Volcano plots showing the comparisons between control and 1 h BCNU/AF treatment in the absence of insulin (d), control and 2 h BCNU/AF treatment in the absence of insulin (e), control and 1 h BCNU/AF treatment in the presence of insulin (f), and basal and insulin-stimulated conditions without BCNU/AF treatment (g). Significantly regulated phosphosites were indicated in red (up-regulated) or blue (down-regulated, adjust $p < 0.05$ and absolute fold change > 1.5).

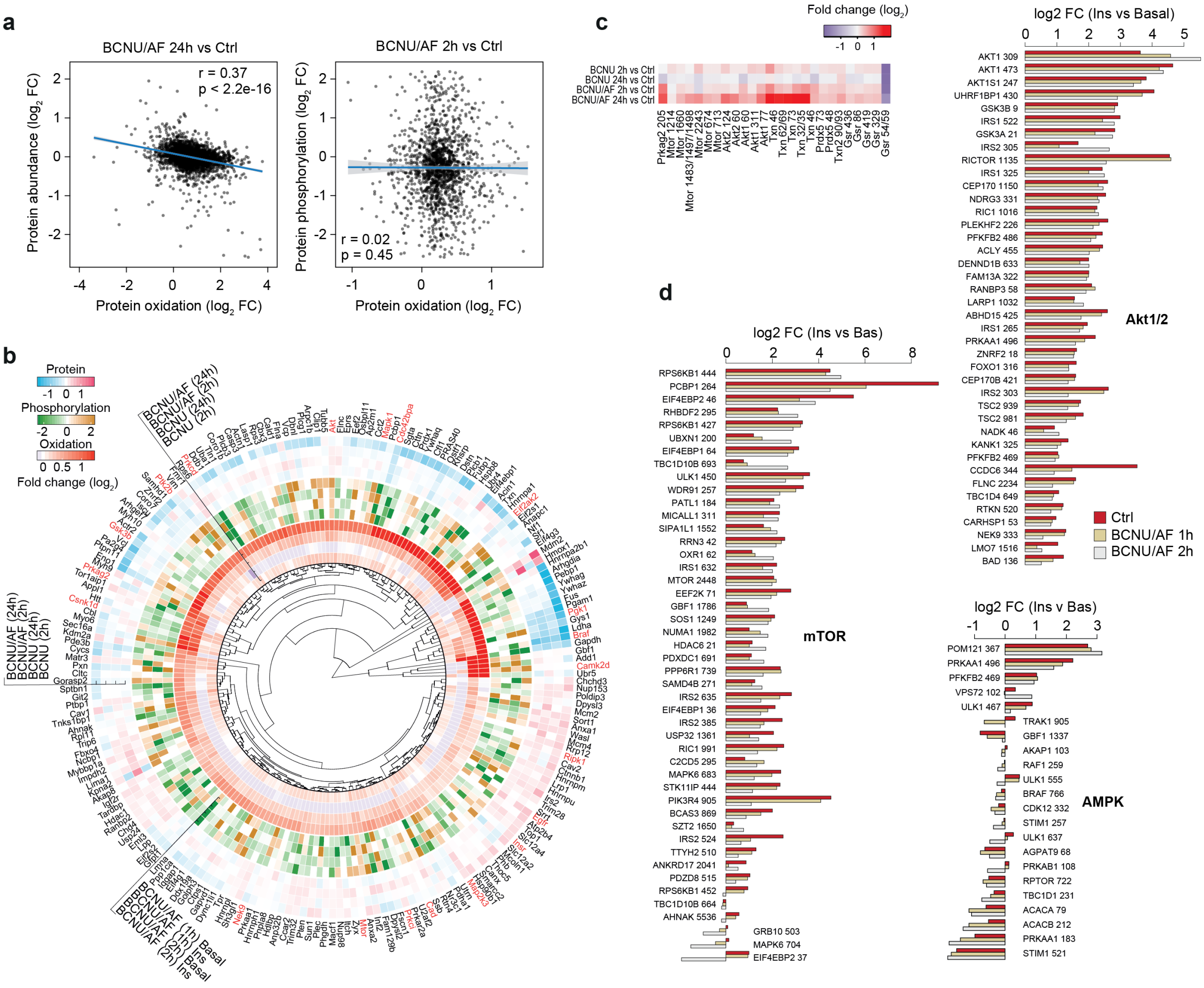

Supplementary Figure 3. Integrative analysis of oxidative stress-regulated phospho-signalling networks. (a) Linear models summarising global relationships of protein oxidation and protein abundance (left panel) and protein oxidation and protein phosphorylation (right panel), respectively, in BCNU/AF treated cells compared to control cells. (b) Redox proteome (inner ring), phosphoproteome (middle ring) and total proteome (outer ring) fold change heatmaps of known kinases (highlighted in red) and substrates that had significantly altered oxidation levels (adjusted *p* < 0.05) after 2 h BCNU/AF treatment. Cysteine site and phosphosite with the largest fold changes of a given protein were used to represent redox proteome and phosphoproteome in a protein-centric manner. Proteins were clustered by their redox proteome. (c) Oxidation levels of Cys sites/peptides in select proteins in control, BCUN, and BCUN/AF treated cells. (d) Phosphorylation levels of Akt, mTOR, and AMPK substrates after insulin stimulation (compared to basal) in WT cells and cells treated with either 1 h or 2 h BCNF/AF.

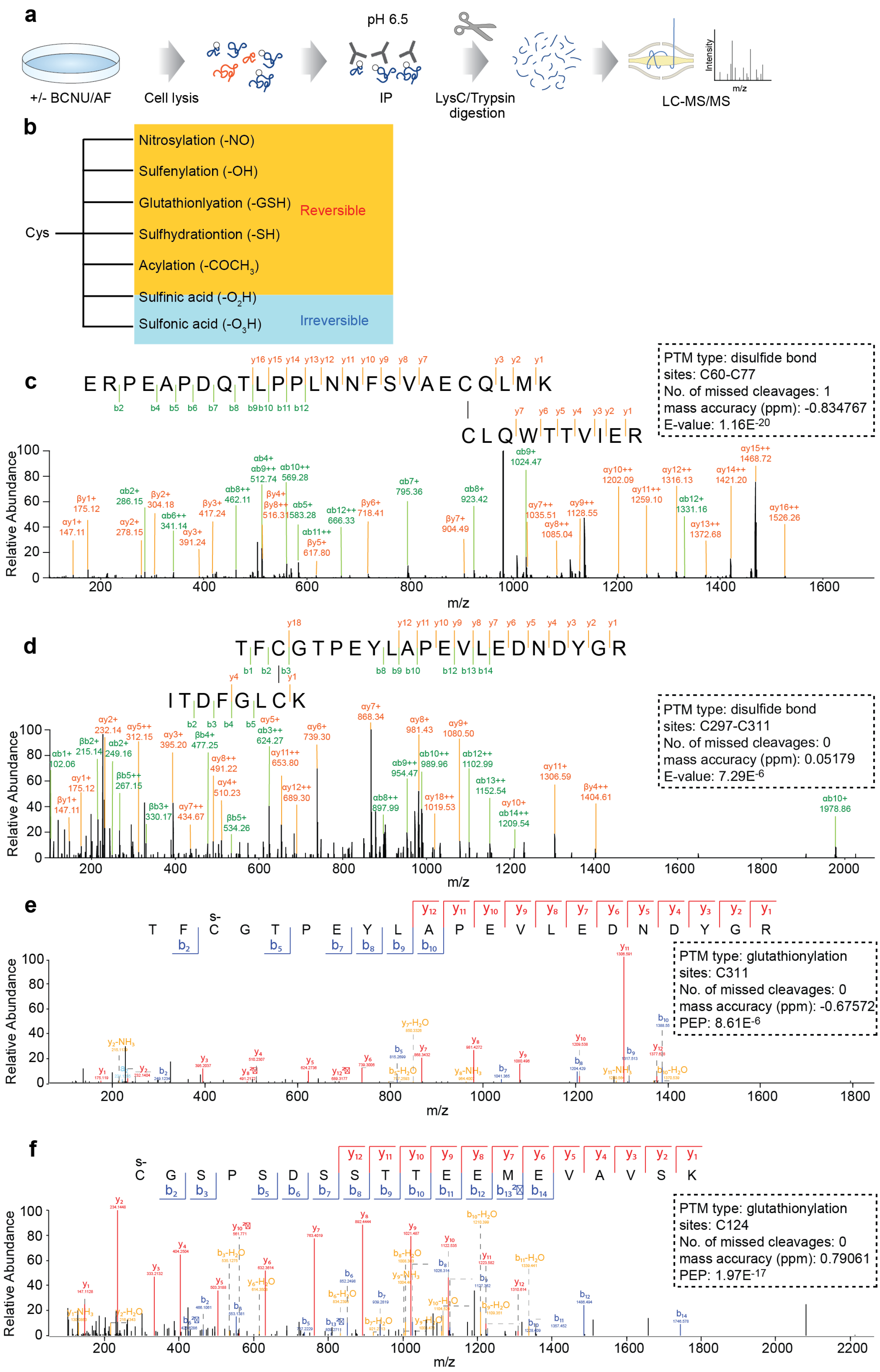

Supplementary Figure 4. Non-reducing IP-MS analysis of Akt2. (a) Experimental design of the non-reducing IP-MS analysis in adipocytes with or without BCNU/AF treatment. (b) Summary of oxidative modifications on cysteine. (c, d) MSMS spectra of identification of disulfide bond C60-C77 (c) and

C297-C311 (d). (e, f) MSMS spectra of identification of glutathionlyation at C311 (e) and glutathionlyation at C124 (f).

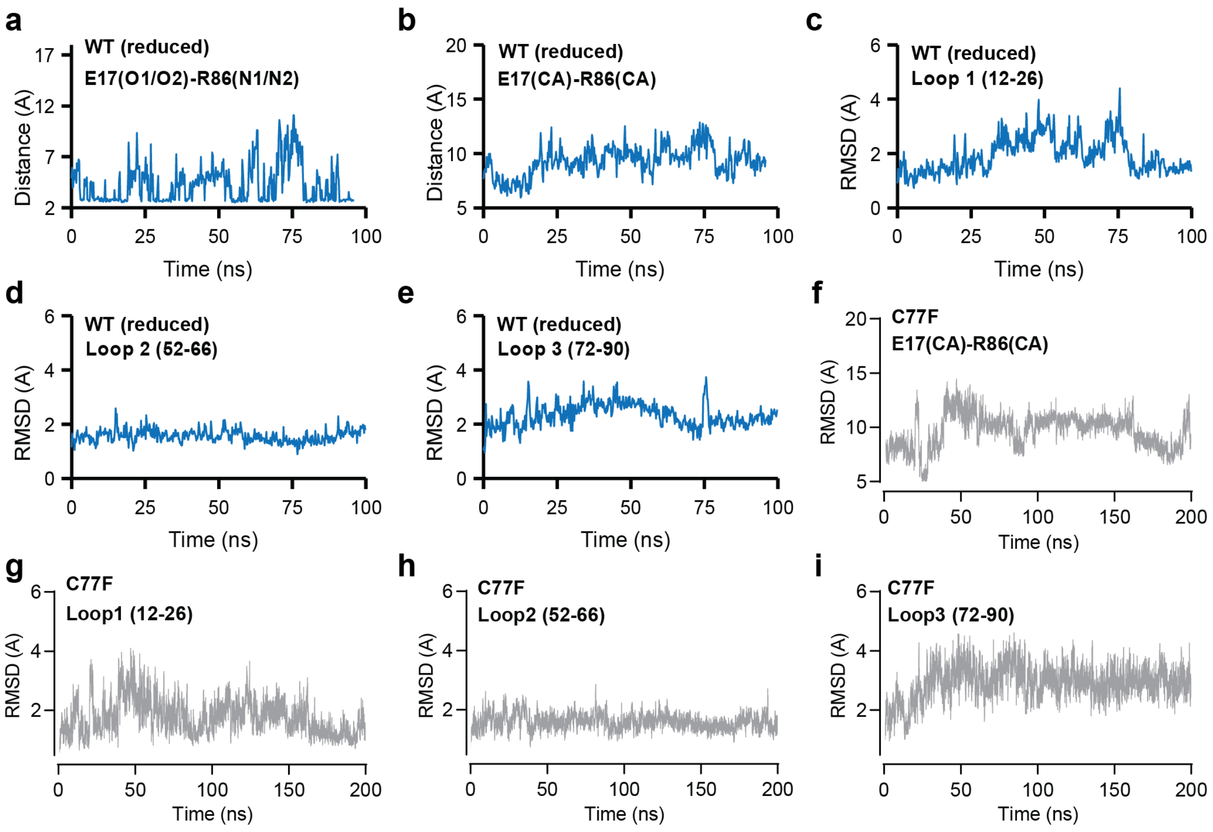

Supplementary Figure 5. Temporal traces of reduced Akt PH domain and the C77F mutant MD simulations. (a) Time series of the E17(O)-R86(N) distance in WT (reduced). Only the distance between the closest O-N atoms are shown. (b) Time series of the Ca-Ca distances between the residues E17 in loop1, and R86 in loop3 in WT (reduced). (c-e) Comparison of the backbone RMSDs of the three loops in WT (reduced) calculated using the crystal structure as a reference. For loop1, the RMSD is calculated for the residues 12-26, which are involved in binding of PIP3**.** (f) Time series of the Ca-Ca distances between the residues E17 in loop1, and R86 in loop3 in C77F. (g-i) Comparison of the backbone RMSDs of the three loops in C77F calculated using the crystal structure as a reference.

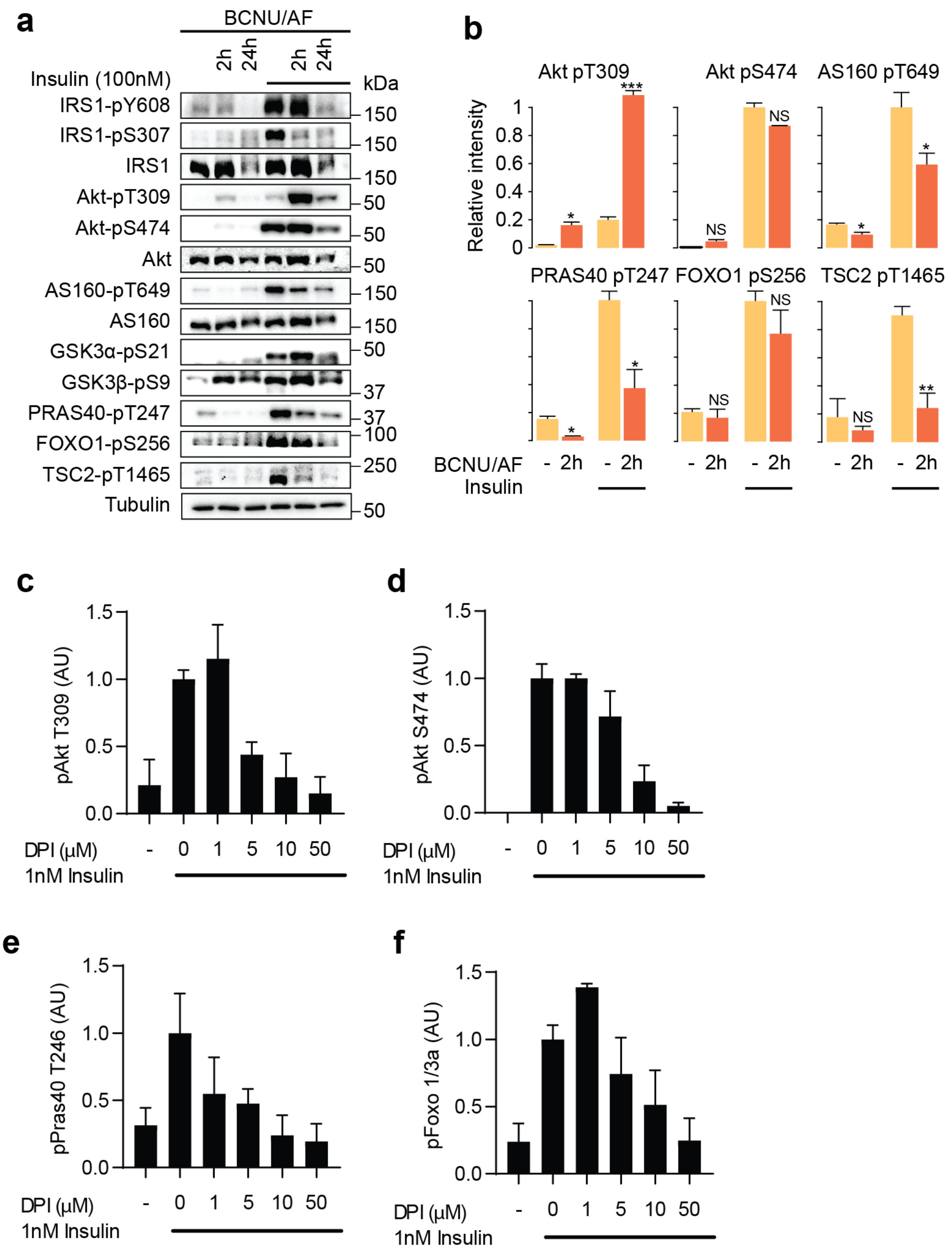

Supplementary Figure 6. A physiological role for the C60/C77 disulfide. (a, b) Quantitation of oxidative stress-regulated Akt signalling by immunoblotting. Adipocytes were treated with BCNU/AF for 2 h, serum starved and stimulated with insulin (100 nM, 20 min). The samples were assessed by immunoblotting with indicated antibodies and quantified from 3 independent experiments. Data are mean ± SEM from n=3, *: $p < 0.05$; **: $p < 0.01$; ***: $p < 0.001$. (c-f) Quantitation of the DPI dose response and its effect on Akt signalling by immunoblotting. Data are mean ± SEM; n=3.

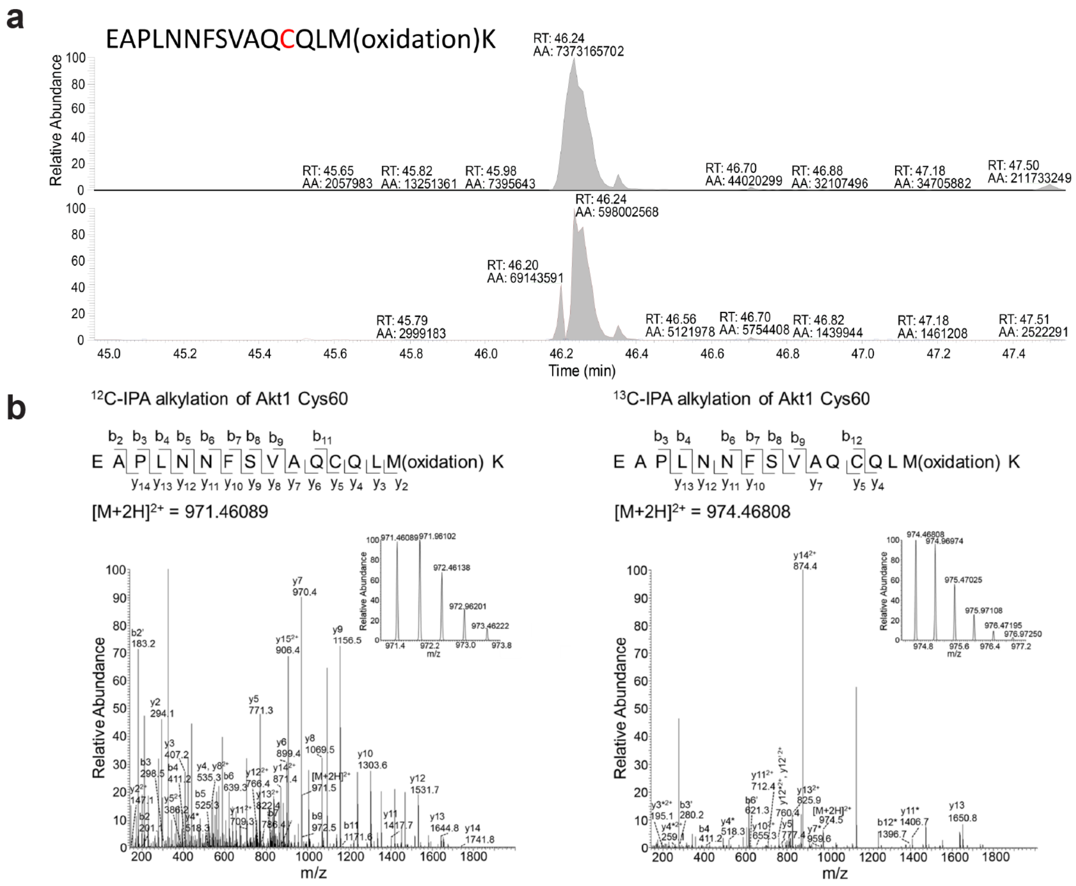

Supplementary Figure 7. Differential cysteine alkylation and mass spectrometry analysis of Akt1 Cys60. (a) HPLC resolution of the Akt1 EAPLNNFSVAQCQLMK peptide containing Cys60 labelled with either 12C-IPA or 13C-IPA. (b) Representative tandem mass spectra of the Akt1 EAPLNNFSVAQCQLMK peptide. At left is an example of 12C-IPA-alkylation of Cys60, and at right is an example of 13C-IPA-alkylation of Cys60. The accurate mass spectrum of the peptide is shown in the inset (left, observed [M+2H]2+ = 971.46089 m/z and expected [M+2H]2+ = 971.4635 m/z; right, observed [M+2H]2+ = 974.46808 m/z and expected [M+2H]2+ = 974.4736).

Supplementary Table 1. List of the interactions in Akt[WT]–PIP3 and Akt[C77F]–PIP3 complexes. The atom-atom contact distances obtained from HADDOCK and MD simulations are listed in units of Å. MD simulations were run for 50 ns after relaxation for each docked system, and the average contact distances were calculated from the last 25 ns. Atom names follow the conventions of the Charmm forcefield.

Akt1	PIP3	Dock [WT]	MD aver. [WT]	PIP3	Dock [C77F]	MD aver. [C77F]
K14 (NZ)	PIP3(OPG/H)	2.6	2.6 ± 0.1	PIP3(O8P)	2.6	2.7± 0.2
K14 (NZ)	PIP3(O8P)	2.4	2.7 ± 0.3	PIP3(O4P)	2.7	2.8± 0.6
E17 (N)	PIP3(OPF/H)	2.7x	2.8 ± 0.2	PIP3(O9P)	2.5	3.1± 0.4
Y18 (N)	PIP3(OPF/H)	3.1	3.7 ± 0.5	PIP3(OPG)	2.6	4.5± 0.7
R23 (N1)	PIP3(O4P/O5P)	2.6	2.7 ± 0.1			
R23 (NE)				PIP3(O9P)	2.5	3.2± 0.6
R25 (NE)	PIP3(O9P)	2.7	2.7 ± 0.1			
R25 (N2)	PIP3(O5P/O6P)	2.9	2.7 ± 0.1	PIP3(OPG/H)	2.6	3.1± 1.5
N53 (ND2)	PIP3(O6P/O8P)	3.2	3.3 ± 0.4	PIP3(O6P)	2.6	2.8± 0.1
R86 (NE)				PIP3(O4P)	2.8	2.7± 0.1
R86 (N2)	PIP3(OPF/G)	2.7	2.7 ± 0.1	PIP3(O5P)	2.5	2.7± 0.1

Supplementary Table 2. Comparison of the replicate simulations for the C77F and C60/77S mutant systems. The average E17(O)–R86(N) and E17(Cα)–R86(Cα) distances, and the average backbone RMSDs for the loop-1 residues 12-26 (in units of (Å) are listed for three simulations. The averages were obtained from the last 100 ns of MD simulations in each case. Atom names follow the conventions of the Charmm forcefield.

Akt	E17(O1/2)–R86(N1/2)	E17(Cα)–R86(Cα)	loop-1 RMSD
Wild type	4.2±1.2<br>7.2±2.9<br>5.8±2.3	7.6±0.9<br>9.7±1.9<br>9.6±1.9	1.0±0.2<br>0.9±0.2<br>1.0±0.2
C77F	2.9±0.9	9.7±1.1	1.5±0.3
	2.8±0.6	10.2±0.6	1.4±0.3
	2.7±0.1	9.8±0.5	1.4±0.2
C60/77S	9.9±2.4	12.7±1.1	3.5±0.6
	8.7±3.0	11.5±1.9	2.8±0.8
	8.4±4.0	12.0±1.3	3.5±0.7

Supplementary Table 3. Reactions and reaction rates of the AKT oxidation-phosphorylation network model.

	Reaction	Reaction rates
R0	Ins+IR ↔ IRa	ka0*Ins*IR - kd0*IRa
R1	PI3K → aPI3K	kc1*PI3K*IRa/(Km1 + PI3K)
R2	aPI3K → PI3K	Vm2*aPI3K
R3	PIP2 → PIP3	kc3*aPI3K*PIP2/(Km3+PIP2)
R4	PIP3 → PIP2	Vm4*PIP3
R5	Akt → Aktox	kc5*ROS*Akt
R6	Aktox → Akt	Vm6*Aktox
R7	PIP3+Aktox ↔ AktoxPM	ka7*PIP3*Aktox - kd7*AktoxPM
R8	Akt + PIP3 ↔ AktPM	ka8*PIP3*Akt - kd8*AktPM
R9	AktPM → AktoxPM	kc5*ROS*AktPM
R10	AktoxPM → AktPM	Vm6*AktoxPM
R11	AktoxPM → Aktox309	kc11*aPDK1*AktoxPM/(Km11+AktoxPM)
R12	Aktox309 → AktoxPM	Vm12*Aktox309
R13	Akt309 →Aktox309	kc5*ROS*Akt309
R14	Aktox309 →Akt309	Vm6*Aktox309
R15	AktPM →Akt309	kc15* aPDK1*AktPM/(Km15+AktPM)
R16	Akt309 →AktPM	Vm16*Akt309
R17	mTORC2 →pmTORC2	kc17*Akt309*mTORC2/(Km17+mTORC2)
R18	mTORC2 →pmTORC2	kc18*Aktox474*mTORC2/(Km18+mTORC2)
R19	pmTORC2 →mTORC2	Vm19*pmTORC2
R20	Aktox309 →Aktox474	kc20*pmTORC2*Aktox309/(Km20+Aktox309)
R21	Aktox474 →Akt + PIP3	Vm21*Aktox474
R22	Akt309 →Akt474	kc22*pmTORC2*Akt309/(Km22+Akt309)
R23	Akt474 →Akt + PIP3	Vm23*Akt474
R24	Akt474 →Aktox474	kc5*ROS*Akt474
R25	Aktox474 →Akt474	Vm6*Aktox474
R26	GSK →pGSK	kc26*Akt474*GSK/(Km26+GSK)
R27	GSK →pGSK	kc27*Aktox474*GSK/(Km27+GSK)
R28	pGSK →GSK	Vm28*pGSK
R29	mTORC2 →pmTORC2	kc29*Aktox309*mTORC2/(Km29+mTORC2)
R30	mTORC2 →pmTORC2	kc30*Akt474*mTORC2/(Km30+mTORC2)
R32	Aktox474 →Aktox309	Vm32*Aktox474
R33	Akt474 →Akt309	Vm33*Akt474
R34	NOX →NOXa	kc34*IRa*NOX/(Km34+NOX)
R35	NOXa →NOX	Vm35*NOXa
R36	∅ → ROS	vs36+kc36a*NOXa/(Km36a+NOXa)/(1+(DPI/Ki36)^3) + kc36b*BCNU/(Km36b+BCNU)
R37	ROS → ∅	(vs36 + kc36a + kc36b)/100*ROS

Supplementary Table 4. Ordinary differential equations of the AKT oxidation-phosphorylation model. The reaction rates are given in Supplementary Table 3.

Left-hand Sides	Right-hand Sides	Initial Conditions (nM)		
		Model 1	Model 2	Model 3
d[IR]/dt	-R0	99.71	99.99	99.84
d[IRa]/dt	R0	0.29	0.01	0.16
d[PI3K]/dt	R2 - R1	100.00	99.56	100.00
d[aPI3K]/dt	R1-R2	0.01	0.44	0.00
d[Akt]/dt	R6 - R5 - R8 + R21 + R23	99.92	1.37	68.24
d[Aktox]/dt	R5 - R6 - R7	0.07	98.62	31.12
d[PIP2]/dt	R4 - R3	99.99	99.83	99.33
d[PIP3]/dt	R3 - R4 - R7 - R8 + R21 + R23	0.00	0.15	0.03
d[AktoxPM]/dt	R7 + R9 - R10 - R11 + R12	0.00	0.00	0.00
d[AktPM]/dt	R8 - R9 + R10 - R15 + R16	0.00	0.00	0.00
d[Aktox309]/dt	R11 - R12 + R13 - R14-R20 + R32	0.00	0.01	0.23
d[Akt309]/dt	R14 - R13 + R15 - R16 -R22 + R33	0.01	0.00	0.26
d[mTORC2]/dt	R19 - R17 - R29 - R18 - R30	99.79	99.09	99.68
d[pmTORC2]/dt	R17 + R29 + R18 - R19 + R30	0.21	0.91	0.32
d[Akt474]/dt	R22 - R33 - R23 - R24 + R25	0.00	0.00	0.15
d[Aktox474]/dt	R20 - R32 - R21 + R24 - R25	0.00	0.00	0.00
d[GSK]/dt	R28 - R26 - R27	93.79	98.73	94.11
d[pGSK]/dt	R26 + R27 - R28	6.21	1.27	5.89
d[ROS]/dt	R36 - R37	0.02	4.15	19.50
d[NOX]/dt	R35 - R34	100.00	100.00	0.00
d[NOXa]/dt	R34 - R35	0.00	0.00	100.00

Supplementary Table 5. Three independent best-fitted parameter sets used for simulations.

Parameter	Value			Unit
	Model 1	Model 2	Model 3	
ka0	109.648	0.363	3.467	nM^-1 min^-1
kd0	380.189	53.703	21.878	Min^-1
kc1	1.380	398.107	7.603	min^-1
Km1	4677.351	114.815	5495.409	nM
Vm2	1.758	2.851	7.244	min^-1
kc3	269.154	0.685	6309.573	min^-1
Km3	8317.638	63.096	0.160	nM
Vm4	1288.250	1.194	691.831	min^-1
kc5	0.396	4073.803	0.115	min^-1
Km5	0.128	19.055	1862.087	nM
Vm6	12.303	234.423	4.887	min^-1
ka7	645.654	0.002	0.682	nM^-1 min^-1
kd7	15.136	0.020	2511.886	nM
ka8	169.824	0.032	0.024	nM min^-1
kd8	147.911	10.233	2290.868	min^-1
kc11	0.830	3.236	16.218	min^-1
Km11	0.027	0.000	0.244	nM
Vm12	0.001	0.002	0.008	min^-1
kc15	9332.543	5011.872	501.187	min^-1
Km15	0.014	295.121	5.035	nM
Vm16	0.366	173.780	0.000	min^-1
kc17	1.050	467.735	0.013	min^-1
Km17	0.009	5.408	6.653	nM
kc18	0.014	11.220	0.003	min^-1
Km18	0.001	1230.269	245.471	nM
Vm19	0.042	0.107	0.023	min^-1
kc20	512.861	30.903	38.019	min^-1
Km20	3981.072	3890.451	44.668	nM
Vm21	0.003	5.309	977.237	min^-1
kc22	6025.596	7.244	6918.310	min^-1
Km22	22.387	0.015	208.930	nM
Vm23	53.703	363.078	1.592	min^-1
kc26	8.590	0.908	3388.442	min^-1
Km26	147.911	12.303	0.662	nM
kc27	1202.264	0.553	7762.471	min^-1
Km27	3630.781	0.242	0.048	nM
Vm28	0.002	0.002	87.096	min^-1
kc29	0.296	81.283	0.003	min^-1
Km29	0.043	2238.721	0.419	nM
kc30	0.112	43.652	0.025	min^-1
Km30	13.490	61.660	0.140	nM
Vm32	3162.278	3.750	891.251	min^-1

Vm33	72.444	4.498	14.454	min^-1
kc34	0.352	5128.614	2818.383	min^-1
Km34	144.544	3235.937	0.000	nM
Vm35	288.403	6456.542	1.368	min^-1
vs36	0.022	0.044	0.001	nM min^-1
kc36a	104.713	0.254	1000.000	nM min^-1
Km36a	44.668	0.114	27.542	nM
kc36b	0.762	0.762	3019.952	nM min^-1
Km36b	0.524	0.004	0.001	nM
Ki36	1.001	1.002	1.010	nM

Supplementary Note 1. Mathematical model description

Implementation

To investigate the network-level dynamics of Akt signalling, we have constructed a new mathematical kinetic model of the AKT signalling network that incorporates the novel oxidation-mediated regulatory mechanisms unveiled from our data. The AKT mathematical model was formulated using ordinary differential equations (ODEs). The model's schematic diagram containing all model reactions is given in Figure 7c. Model ODEs, rate equations and the sets of best-fitted parameter values used for simulations are given in Supplementary Tables 3-5. The model was implemented and numerically simulated in MATLAB ® (The MathWorks. Inc. 2018b) and IQM Tools (http://www.intiquan.com/intiquan-tools/). IQM Tools was used to convert a standard IQM model into C code, which is compiled to an executable MEX file for model simulation.

Model fitting (calibration)

The adequacy of a mathematical model is generally justified by its ability to recapitulate known experimental data, which is ensured through a process known as model fitting or calibration where unmeasured model parameters are numerically estimated so that model simulations fit the data. Parameter estimation was done by minimizing the following 'objective function' that quantifies the discrepancy between simulated values and corresponding experimental measurements:

$$J(\boldsymbol{p}) = \sum_{j=1}^{M} \sum_{i=1}^{N} \left(\frac{y_{j,i}^{D} - y_j(t_i, \boldsymbol{p})}{\sigma_{j,i}} \right)^2 \qquad (1)$$

where M is the number of the given experimental data sets used for fitting and N is the number of time points within each experimental data set. $y_j(t_i, \boldsymbol{p})$ represents the numerical solution for the model state variable y_j evaluated at time t_i and parameter set $\boldsymbol{p}$; while $y_{j,i}^{D}$ is the mean value of the corresponding data point at t_i with the associated error variance $\sigma_{j,i}$.

A Genetic Algorithm (GA) was used to optimize the objective function [1-3]. This was done by using the Global Optimization Toolbox and the function *ga* in MATLAB. Selection rules select the individual solutions with the best fitness values (called 'elite solution') from the current population. The elite count was set to 5% of the population size. Crossover rules combine two parents to generate offspring for the next generation. The crossover faction was set at 0.8. Mutation rules apply random changes to individual parents to generate the population of the next generation. For the mutation rule, we generated a random number from a Gaussian distribution with mean 0 and standard deviation σ_k, which was applied to the individuals of the current generation. The standard deviation function (σ_k) is given by the recursive formula as follows:

$$\sigma_k = \sigma_{k-1}\left(1 - \frac{k}{G}\right), \qquad (2)$$

where k is the kth generation, G is the number of generation, and $\sigma_0 = 1$.

To derive at the best fitted parameter set, we carried out repeated GA runs with population size of 500 and the generation number set to 100. In this computation, we also changed the mutation and crossover rates and even the population size to escape from being trapped in local minima. After multiple repetitions of the GA process where the best fitted set obtained from a previous repeat was used as the starting point of the next repeat, we arrived at the final best fitted set as the objective function was not further reduced, and the fitted parameter values no longer change. This whole process was repeated 3 times to obtain 3 independent equally best-fitted parameter sets (Supplementary Table 5) which was subsequently used for simulations.

Key model assumptions

Binding of insulin (Ins) to the insulin receptor (IR) induces IR phosphorylation and the subsequent recruitment of effector proteins including the IRS family proteins. These molecules undergo phosphorylation and form a complex with and activates PI3K. To keep the model simple without compromising its dynamic accuracy, we simply modelled this cascade as a one-step PI3K activation (reactions 1-2, Figure 7a). We also assumed that ROS can be induced by IR through a plasma membrane coupled NADPH oxidase (NOX) [4] (reactions 34-35). PIP3 produced by PI3K recruits AKT to the plasma membrane, which is assumed to be reversible (reaction 3&4). Importantly, we have identified two cysteine residues C60 and C77 in the pleckstrin homology (PH) domain of AKT, and shown that oxidised AKT displays stronger binding to PIP3, which is reflected by a higher binding affinity of oxidised AKT to PIP3 (reaction 7). We assumed that ROS promotes oxidation of various moieties of cytoplasmic as well as membrane-bound AKT (reactions 5&6, 9&10, 13&14, 24&25). Moreover, the oxidized (-ox) and reduced (-red) AKT species at the plasma membrane were assumed to be phosphorylated at T309 (reaction 11 & 15) and primed for the further activation by mTORC2 on S474. Importantly, the phosphorylated Akt activates mTORC2, which forms a positive feedback loop [5].

Supplementary References

1. Reali, F., C. Priami, and L. Marchetti, *Optimization Algorithms for Computational Systems Biology.* Frontiers in Applied Mathematics and Statistics, 2017. **3**(6).
2. Man, K.F., K.S. Tang, and S. Kwong, *Genetic algorithms: concepts and applications [in engineering design].* IEEE Transactions on Industrial Electronics, 1996. **43**(5): p. 519-534.
3. Shin, S.Y., et al., *The switching role of b-adrenergic receptor signalling in cell survival or death decision of cardiomyocytes.* Nat Commun, 2014. **5**: p. 5777.
4. Goldstein, B.J., et al., *Role of insulin-induced reactive oxygen species in the insulin signaling pathway.* Antioxid Redox Signal, 2005. **7**(7-8): p. 1021-31.
5. Yang, G., et al., *A Positive Feedback Loop between Akt and mTORC2 via SIN1 Phosphorylation.* Cell Rep, 2015. **12**(6): p. 937-43.
