# Supplementary material for: Global redox proteome and phosphoproteome analysis reveals redox switch in Akt
Source: Nat Commun. 2019 Dec 2;10:5486. doi: 10.1038/s41467-019-13114-4 (PMC6889415; doi:10.1038/s41467-019-13114-4)
Supplement: Supplementary file 3 — Description of Additional Supplementary Files [file 41467_2019_13114_MOESM3_ESM.docx]

**Description of Supplementary Files**

**File Name: Supplementary Data 1**

**Description:** Identification of 13,451 oxidized peptides.

**File Name: Supplementary Data 2**

**Description:** Uniprot database annotation of 1,550 oxidized cysteine sites.

**File Name: Supplementary Data 3**

**Description:** Quantification of 7,451 proteins.

**File Name: Supplementary Data 4**

**Description:** DE analysis of total proteome.

**File Name: Supplementary Data 5**

**Description:** DE analysis and direction-based integrative analysis of redox proteome.

**File Name: Supplementary Data 6**

**Description:** Identification of 23,647 phosphosites.

**File Name: Supplementary Data 7**

**Description:** DE analysis of phosphoproteome.

**File Name: Supplementary Data 8**

**Description:** Comparison of the average distances (in Å) between the interacting Akt1 residues and PIP3 sites obtained from the crystal structure 1UNQ with those obtained from the MD simulations of the WT and mutant Akt1.
