# Supplementary material for: Global redox proteome and phosphoproteome analysis reveals redox switch in Akt
Source: Nat Commun. 2019 Dec 2;10:5486. doi: 10.1038/s41467-019-13114-4 (PMC6889415; doi:10.1038/s41467-019-13114-4)
Supplement: Supplementary file 12 — Reporting Summary [file 41467_2019_13114_MOESM12_ESM.pdf]

nature research

Corresponding author(s): David E. James

Last updated by author(s): Oct 9, 2019

Reporting Summary

Nature Research wishes to improve the reproducibility of the work that we publish. This form provides structure for consistency and transparency in reporting. For further information on Nature Research policies, see Authors & Referees and the Editorial Policy Checklist .

Statistics

For all statistical analyses, confirm that the following items are present in the figure legend, table legend, main text, or Methods section.

n/a	Confirmed	
☐	☒	The exact sample size (*n*) for each experimental group/condition, given as a discrete number and unit of measurement
☐	☒	A statement on whether measurements were taken from distinct samples or whether the same sample was measured repeatedly
☐	☒	The statistical test(s) used AND whether they are one- or two-sided<br>*Only common tests should be described solely by name; describe more complex techniques in the Methods section.*
☒	☐	A description of all covariates tested
☐	☒	A description of any assumptions or corrections, such as tests of normality and adjustment for multiple comparisons
☐	☒	A full description of the statistical parameters including central tendency (e.g. means) or other basic estimates (e.g. regression coefficient) AND variation (e.g. standard deviation) or associated estimates of uncertainty (e.g. confidence intervals)
☐	☒	For null hypothesis testing, the test statistic (e.g. *F*, *t*, *r*) with confidence intervals, effect sizes, degrees of freedom and *P* value noted<br>*Give P values as exact values whenever suitable.*
☒	☐	For Bayesian analysis, information on the choice of priors and Markov chain Monte Carlo settings
☐	☒	For hierarchical and complex designs, identification of the appropriate level for tests and full reporting of outcomes
☐	☒	Estimates of effect sizes (e.g. Cohen's *d*, Pearson's *r*), indicating how they were calculated

Our web collection on statistics for biologists contains articles on many of the points above.

Software and code

Policy information about availability of computer code

Data collection

MS-based redox proteomic analysis was performed on an Easy nLC-1000 UHPLC coupled to a Q-Exactive mass spectrometer in positive polarity mode. Peptides were separated using an in-house packed 75 µm x 40 cm column (1.9 µm particle size, ReproSil Pur C18-AQ) with a gradient of 5-30% ACN containing 0.1% FA over 90 min at 200 nL/min at 55 ºC. The MS1 scan was acquired from 300-1,750 m/z (70,000 resolution, 3e6 AGC, 100 ms injection time) followed by MS/MS data-dependent acquisition of the top 20 ions with HCD (17,500 resolution, 5e5 AGC, 60 ms injection time, 30 NCE, 2.0 m/z isolation width).

Phosphopeptides were separated by HPLC in a single run (without pre-fractionation) and analyzed on a Q Exactive HF-X mass spectrometer. Peptides were separated using an in-house packed 75 µm x 40 cm column (1.9 µm particle size, ReproSil Pur C18-AQ) with a gradient of 3-41% ACN containing 0.1% FA over 90 min at 350 nL/min at 55 ºC. The MS1 scan was acquired from 300-1,600 m/z (60,000 resolution, 3e6 AGC, 120 ms injection time) followed by MS/MS data-dependent acquisition of the top 10 ions with HCD (15,000 resolution, 1e5 AGC, 50 ms injection time, 27 NCE, 1.6 m/z isolation width).

Total proteome MS analysis was performed on the same LC/MS system including an Easy nLC-1000 UHPLC coupled to a Q-Exactive mass spectrometer with the same method as redox proteomic analysis except that NCE was set to 27.

Non-reducing IP-MS analysis was performed on the same LC/MS system including an Easy nLC-1000 UHPLC coupled to a Q-Exactive mass spectrometer with the same method as redox proteomic analysis except that NCE was set to 25.

Microscopy was performed on an inverted Nikon TiE with H-TIRF. Okolabs incubator was maintained at 37C for all experiments. TIRF was introduced at an angle corresponding to a penetration depth of ~70-90 nm. All data were acquired with Nikon Elements software. MD simulations were performed using the NAMD program 74 with the CHARMM36 force field 75. An NPT ensemble with the periodic boundary conditions was employed in the MD simulations. The pressure and temperature were maintained at 1 atm and 300 K, respectively, via Langevin coupling with damping coefficients of 5 ps−1. Lennard-Jones interactions were switched off smoothly within a distance of 10–13.5 Å, and the particle-mesh Ewald algorithm were applied to calculate the long-range electrostatic interactions. A time step of 2 fs was used to generate the trajectory data in MD simulations.

The simulation systems of Akt1 PH domain for both WT and mutant were prepared using the VMD software 73. Water box size for both systems were selected so that there were at least three layers of waters between the protein and box boundaries. The systems were ionized and neutralized with 0.1 M of KCl solution. In MD simulations, the systems were first equilibrated with restrained protein atoms at 300 K to obtain the correct water density with 1 atm pressure coupling. Then the side chain and backbone atoms were simultaneously

	relaxed in several steps from k = 5 kcal/mol/Å2 to 0, simulating the systems for 200 ps at each step. Production data were generated from 100 ns (for WT vs C60/77S) and 200ns (for C77F).
Data analysis	Redox proteome, phosphoproteome and total proteome raw data was processed using MaxQuant (v1.6.1.2); Disulfide bonds were identified using pLink-SS; For the identification of the other reversible oxidative modifications on cysteines, raw data was processed using MaxQuant (v1.5.2.10). Data analyses were performed using R programming environment (3.4.3). Phosphosites were analysed using directPA 1.3 (https://CRAN.R-project.org/package=directPA). Limma 3.32.2 (https://bioconductor.org/packages/relsease/bioc/html/limma.html) was used for differential analysis. Pathway analysis was performed using DAVID 70 (v6.8 beta, released in May/2016, https://david-d.ncifcrf.gov/).Image data were analysed using Fiji. Graphs and statistical analyses were performed with either R or Graphpad Prism 7.02. MD simulations of the proteins, and the trajectory data were analysed using the built-in functions of VMD.

For manuscripts utilizing custom algorithms or software that are central to the research but not yet described in published literature, software must be made available to editors/reviewers. We strongly encourage code deposition in a community repository (e.g. GitHub). See the Nature Research guidelines for submitting code & software for further information.

Data

Policy information about availability of data

All manuscripts must include a data availability statement. This statement should provide the following information, where applicable:

- Accession codes, unique identifiers, or web links for publicly available datasets
- A list of figures that have associated raw data
- A description of any restrictions on data availability

All MS data have been deposited to the ProteomeXchange Consortium via the PRIDE partner repository with the dataset identifier PXD011525.

Field-specific reporting

Please select the one below that is the best fit for your research. If you are not sure, read the appropriate sections before making your selection.

- [x] Life sciences
- [ ] Behavioural & social sciences
- [ ] Ecological, evolutionary & environmental sciences

For a reference copy of the document with all sections, see nature.com/documents/nr-reporting-summary-flat.pdf

Life sciences study design

All studies must disclose on these points even when the disclosure is negative.

Sample size	Samples for mass spectrometry-based analysis were performed with three or four biological replicates.
Data exclusions	No data were excluded from the analyses.
Replication	All data were successfully reproduced. All MS experiments were performed as triplicates or quadriplicates as a minimum. Imaging data were obtained from a minimum of 3 independent experiment. Most biological experiments were carried out at least 3 times and experimental findings were reliably reproduced.
Randomization	Randomization/blinding was not applicable to the study.
Blinding	Randomization/blinding was not applicable to the study.

Reporting for specific materials, systems and methods

We require information from authors about some types of materials, experimental systems and methods used in many studies. Here, indicate whether each material, system or method listed is relevant to your study. If you are not sure if a list item applies to your research, read the appropriate section before selecting a response.

Materials & experimental systems

n/a	Involved in the study	
[ ]	[x]	Antibodies
[ ]	[x]	Eukaryotic cell lines
[x]	[ ]	Palaeontology
[ ]	[x]	Animals and other organisms
[x]	[ ]	Human research participants
[x]	[ ]	Clinical data

Methods

n/a	Involved in the study	
[x]	[ ]	ChIP-seq
[x]	[ ]	Flow cytometry
[x]	[ ]	MRI-based neuroimaging

Antibodies

Antibodies used	anti-PRDX2 (Abcam, ab71533); anti-PRDX3 (Abfrontier, BS-1874R); anti-AKT-pT309 (CST, 9275); anti-AKT-pS474 (CST, 4051); anti-AKT2 (CST, 3063); anti-AS160-pT642 (CST, 4288); anti-TSC2-pT1462 (CST, 3611); anti-PRAS40-pT247 (CST, 2997); anti-GSK3a-pS21 (CST, 9327); anti-IRS1-pS307 (CST, 2381); anti-FOXO1-pT256 (CST, 9461); anti-mTOR-pS2448 (CST, 5336); anti-p70S6K-pT390 (CST, 9234); anti-p70S6K-pT421/S424 (CST, 9204); anti-RICTOR-pT1135 (CST, 3806); anti-S6-pS240/S244 (CST, 2215); anti-AMPKa-pT183 (CST, 2535); anti-ACC-pS79 (CST, 3661); anti-dAKT-pS505 (CST, 4054); anti-Tubulin (CST, 5335); anti-Tubulin (CST, 5335); anti-Actin (CST, 4967); anti-RFP (Life technologies, R10367); anti-FLAG (Sigma, F3165); anti-14-3-3 (Santa Cruz, sc-1657); all the other antibodies are home made.
Validation	Most of the antibodies used in this research were from commercial sources, and antibodies were validated per manufacturers instructions.

Eukaryotic cell lines

Policy information about cell lines

Cell line source(s)	3T3L1's were obtained from Howard Green at Harvard; HEK293 and HeLa cells were purchased from ATCC.
Authentication	No further Authentication has been performed.
Mycoplasma contamination	All cells were routinely tested for Mycoplasma and confirmed free of contamination
Commonly misidentified lines (See ICLAC register)	N/A

Animals and other organisms

Policy information about studies involving animals; ARRIVE guidelines recommended for reporting animal research

Laboratory animals	Drosophila melanogaster
Wild animals	n/a
Field-collected samples	n/a
Ethics oversight	This is not required for flies.

Note that full information on the approval of the study protocol must also be provided in the manuscript.
